# Cancer Risk Behaviors, Cancer Beliefs, and Health Information Seeking Among Under-Represented Populations in San Francisco: Differences by Sexual Orientation and Gender Identity

**DOI:** 10.1089/heq.2022.0013

**Published:** 2022-09-01

**Authors:** Eduardo J. Santiago-Rodríguez, Natalie A. Rivadeneira, Michelle A. DeVost, Urmimala Sarkar, Robert A. Hiatt

**Affiliations:** ^1^ Department of Epidemiology and Biostatistics, University of California, San Francisco, San Francisco, California, USA.; ^2^ Department of Epidemiology, University of North Carolina Gillings School of Global Public Health, Chapel Hill, North Carolina, USA.; ^3^ Division of General Internal Medicine, Department of Medicine, University of California, San Francisco, San Francisco, California, USA.

**Keywords:** sexual orientation and gender identity, sexual and gender minority, health information seeking, cancer risk behaviors, cancer beliefs, San Francisco

## Abstract

**Purpose:**

Sexual and gender minority (SGM) individuals in the United States are at increased risk of cancer compared to the non-SGM population. Understanding how SGM persons perceive cancer risk and their practices and preferences for accessing health information is key for improving the preventive and health care services they receive.

**Methods:**

In this cross-sectional study, we analyzed data from the San Francisco Health Information National Trends Survey. SGM individuals were identified by self-report. Differences in cancer risk factors, cancer beliefs, and health information seeking were evaluated by SGM status using multivariable logistic regression models.

**Results:**

Out of 1027 participants, 130 (13%) reported being SGM individuals. Current smoking (odds ratio [OR]=1.93, 95% confidence interval [CI]=1.24–3.01) and alcohol use (OR=1.69, 95% CI=1.10–2.59) were more common among SGM persons than among non-SGM persons. No differences by SGM status were observed in health information seeking behaviors, preferences, and cancer beliefs, but SGM participants reported significantly higher odds of feeling frustrated (OR=1.78, 95% CI=1.20–2.64) and having concerns about the quality of the information (OR=1.54, 95% CI=1.03–2.31) during their most recent health information search.

**Conclusions:**

Intervention efforts aimed at SGM individuals with current use of tobacco and/or alcohol should be expanded. SGM communities also need improved access to consistent, reliable, and accurate sources of health information. Their increased frustration when seeking health information and concerns about the quality of the information they find have important implications for SGM health and care, and the drivers of these differences merit further evaluation.

## Introduction

In the United States, it is estimated that 7.1% of the adult population (or ∼18.3 million adults) are sexual and gender minority (SGM) individuals.^1^ Yet, most national health surveillance systems have not always collected sexual orientation information, and gender identity data remain limited or nonexistent.^2–4^ The lack of accurate sexual orientation and gender identity (SOGI) information in health data sources reflects the systemic exclusion and marginalization that SGM people continue to face, and this exclusion has consequences for their health and wellbeing.^4^

Although population-level data about health outcomes stratified by SOGI in the United States are scarce, many reports indicate that SGM persons have higher incidence of many diseases and higher mortality than the general US population.^5–10^ The absence of high-quality information about SOGI in national surveys and other data sources contributes to these disparities and impedes the observation of evidence necessary for the development of public health interventions to improve SGM health.^11^

**FIG. 1.**
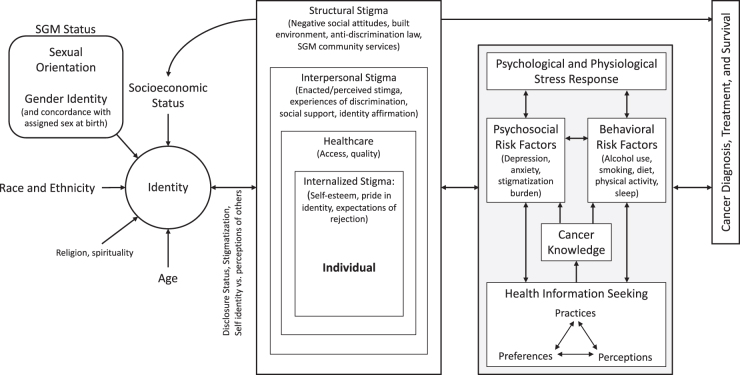
Conceptual model of the determinants of cancer outcomes. Adapted from the American Heart Association's Intersectional Transgender Multilevel Minority Stress Model.

SGM persons are at increased risk of chronic diseases, including cancer, as a result of systemic stigmatization, discrimination, and experiences of minority stress (Fig. 1).^5^ Chronic exposure to stress across the life span has been linked to a greater prevalence of high-risk behaviors (e.g., physical inactivity and the use of tobacco products and alcohol) in SGM individuals.^9,12–16^ Intersectionality theory tells us that individuals with multiple marginalized identities—such as those based on race or ethnicity, religion, human immunodeficiency virus (HIV) status, poverty, disability, or other personal attributes—are at highest risk of stigmatization burden and associated psychological and physiological stressors.^17–19^

They are subsequently the least likely to receive adequate care across the cancer continuum compared to nonmarginalized individuals and other SGM persons leading to additional burden on multiple marginalized SGM individuals.^7,18,20–23^ SGM adults in the United States are also disproportionately likely to have HIV, putting them at much higher risk of developing many types of cancer.^24^ Barriers to receipt of optimal cancer care among SGM persons include lack of knowledge about cancer risk factors and screening guidelines, inaccurate cancer beliefs, poor clinician–patient communication, providers' limited knowledge of SGM health and their specific needs, and stigma and discrimination at individual, interpersonal, and structural levels.^5,25–27^

Understanding how SGM persons perceive cancer risk and their practices and preferences for accessing health information is key for improving the health care services they receive and reducing health inequities in this group. Prior literature on these topics among SGM individuals in the United States have often used national population-based surveys (e.g., Health Information National Trends Survey [HINTS], Behavioral Risk Factor Surveillance System [BRFSS], National Health Interview Survey [NHIS]), that—despite being rigorously collected, high-quality data sources—suffer two major limitations for studying SGM health. First, the questions used to identify SGM individuals in these surveys do not follow best practice recommendations for the collection of SOGI data, which may lead to misclassification of SGM status and biased results.^2,3,28,29^

Second, although these survey samples are designed to be representative of the US population, SGM persons—particularly those with multiple minoritized identities—are less likely to be included in these study populations.^29^ Study participation often requires individuals to own a phone or to reside in traditional housing units, so SGM individuals from racial and ethnic minoritized groups, those with low socioeconomic status (SES), those who are undocumented, and those who are unhoused are more likely to be excluded from these samples than those who are White, stably housed, and have high-SES.

Consequently, the most privileged SGM individuals are over-represented in these data, and their findings are generalized to all SGM persons.^29^ A strategy that has been proposed to overcome this limitation is to target the population of interest through community recruitment with purposive/selective sampling approaches, in which researchers recruit individuals with specific characteristics to facilitate research among hard-to-reach and hard-to-identify populations.^5,28–30^

A recent analysis of Gallup survey data from 2012 to 2017 estimated that 6.7% of people residing in the San Francisco Bay Area identified as SGM individuals, the highest proportion of any large metropolitan area in the United States.^31^ San Francisco's population is also characterized by its racial and ethnic diversity and by its socioeconomic heterogeneity.^32^ Leveraging these regional characteristics, research that employs purposive sampling methods to ensure the participation of marginalized populations and that follows best practices for measuring SOGI data offers a unique opportunity for understanding inequities in SGM health. In this study, we used a community-based sampling strategy and targeted areas in San Francisco where racially and ethnically minoritized groups are predominant (i.e., non-Hispanic Black, Hispanic, and non-Hispanic Asian/Pacific Islander), where non-English language speakers (i.e., Spanish, Cantonese, and Mandarin) are prevalent, and where housing insecurity is evident.

We emphasized these neighborhood characteristics to oversample more vulnerable populations, who are also the most likely to be missed in national samples. The objective of this study was to quantify the relationship between SGM status (as a proxy for experiences of SOGI-based stigma and discrimination against SGM individuals) and cancer risk behaviors, cancer beliefs, and health information seeking behaviors, preferences, and perceptions in a multiethnic urban population. Findings from this study will inform future interventions directed to improve cancer prevention and early detection practices in SGM communities.

## Methods

### Data source, study design, and study population

In this cross-sectional study we analyzed data from the San Francisco Health Information National Trends Survey (SF-HINTS). SF-HINTS was conducted in 2017 as part of the research activities of the San Francisco Cancer Initiative (SF CAN), a collaborative effort between health care providers, academic centers, government, community groups, and residents to reduce the burden of cancer in the city.^33^

Unlike HINTS, which employs a two-stage sampling design to recruit a representative sample of the United States, SF-HINTS used community-based snowball sampling to reach members of diverse populations often excluded from research.^30,34^ SF CAN established collaborations with community-based organizations serving the target populations, and specific recruitment sites were identified.^35^ Potential study participants were reached at popular community locations in San Francisco (e.g., street markets, parks, community events) between May and September 2017. Individuals were included in the study if they resided in the City and County of San Francisco (based on the zip codes they provided), were 18–75 years old, and were able to complete the survey in English, Spanish, Mandarin, or Cantonese.

Prespecified proportions of the total sample were recruited according to two characteristics of interest: preferred language and race/ethnicity. Fifty-percent of all interviews were conducted in English (among those, 50% of respondents were African American) and the remainder in Cantonese or Mandarin (25%) and Spanish (25%). Bilingual and bicultural members of the research team obtained informed consent and administered surveys face-to-face using iPads. Whenever participants were comfortable, they were able to complete the survey independently under the supervision of the survey administrator. Data were collected using the Research Electronic Data Capture (REDCap) web-based application.^36,37^ The Internal Review Board of the University of California San Francisco approved this study (number: 16-20707).

### Measures

Participants were considered SGM persons based on self-report to the following SOGI questions: “What is your current gender identity?,” “What sex were you assigned at birth on your original birth certificate?,” and “How do you describe your sexual orientation or sexual identity?.” A person was classified as SGM if their reported gender identity was “Female-to-Male/Transgender Male/Trans Man,” “Male-to-Female/Transgender Female/Trans Woman,” “Genderqueer,” any response other than “Male” and “Female,” or had discordant answers with respect to “Male” and “Female” in the first two questions and/or if their reported sexual orientation was “Gay/Lesbian/Same-Gender Loving,” “Bisexual” or any response other than “Straight/Heterosexual.”

These SOGI questions were taken from the guidelines developed by the San Francisco Department of Public Health, which are based on best practices for measuring and identifying SGM people as outlined by the Committee on Lesbian, Gay, Bisexual, and Transgender Health Issues and Research Gaps and Opportunities of the Institute of Medicine.^6,38^ For analyses, all SGM groups were combined and compared to non-SGM individuals.

The outcome variables in this study fell into two domains: (1) health information seeking behaviors, preferences, and perceptions, and (2) cancer risk behaviors and beliefs. Questions about health information seeking behaviors, preferences, and perceptions included whether they have ever looked for information about health or medical topics from any source, their preferred source of information, their preferences for getting information from providers, sources used for getting health information from a provider in the last year, and their perceptions during their most recent health information search.

To assess cancer risk behaviors, respondents were asked about lifetime smoking of at least 100 cigarettes (yes/no), current smoking (yes/no), use of e-cigarettes or other electronic smoking devices (yes/no), alcohol use (dichotomized for analysis as either non-use/once-a-year use or everyday/weekly/monthly use of any beverage containing alcohol) and physical inactivity (“any physical activities or exercises such as running, golf, gardening, or walking for exercise,” yes/no).

To assess cancer beliefs, a Likert-type scale was used to measure participants' level of agreement (strongly agree, somewhat agree, somewhat disagree, and strongly disagree) with the following five statements: “Everything causes cancer,” “There is not much you can do to lower your chances of getting cancer,” “There are so many recommendations about preventing cancer that it is hard to know which ones to follow,” “Cancer is most often caused by behavior or lifestyle,” and “When I think about cancer, I think about death.” For analysis, responses were collapsed into two categories (agree, disagree).

Additional sociodemographic and health care access variables were collected and considered in the analysis. These included age (continuous), race/ethnicity (Non-Hispanic White, Non-Hispanic Black, Hispanic, non-Hispanic Asian/Pacific Islander, and Other), marital status (dichotomized as single/divorced/separated/widowed or married/domestic partnership/living as married), education (less than high school, completed high school, and more than high school), employment status (employed, unemployed, retired, and disabled), household income (<$10k, $10k to <$20k, $20k to <$50k, and ≥$50k), housing status (stable or unstable) and place of birth (US or foreign-born).

The health care access variables measured were health insurance coverage (Medicaid, Medicare, private, other, or none), having usual place of care (yes/no), and times received care in the last year (0, 1–2, 3–4, and more than 4). All questions for the outcomes and additional covariates were taken from the repository of validated questions featured in HINTS 5, Cycle 1 and the 2016 BRFSS survey.^39,40^

### Statistical analysis

Descriptive analyses were conducted to summarize and compare the characteristics of the population under study by SGM status. Differences in sociodemographic characteristics and health care access were evaluated using Student's *t*-test, Pearson's chi-squared test, or Fisher's exact test. Unadjusted and adjusted logistic regression models were fitted to assess the association between SGM status and the outcomes of interest. Since the relationship between SOGI-based stigma and discrimination and any of the outcomes under study could plausibly be confounded by other sociodemographic variables, we included age, race/ethnicity, and education (a proxy for SES) as covariates in our multivariable regression models. Regression results are reported as odds ratios (ORs) with 95% confidence intervals (95% CIs). For questions in which participants answered “do not know” or refused to answer, the information was treated as missing data and excluded from the analysis. All analyses were conducted in Stata (Version 15.1, College Station, TX).

## Results

In SF-HINTS, a total of 1027 participants were surveyed, and among them 130 (13%) were classified as SGM individuals (lesbian/gay, *n*=64; bisexual, *n*=39; transgender, *n*=14; queer/other, *n*=13). Compared to non-SGM, SGM persons were significantly younger and more educated. Also, a significantly higher proportion of SGM participants reported being Hispanic ethnicity, not having a partner, being unemployed, and experiencing housing instability in the last year (Table 1).

**Table 1.** Characteristics of Participants in the San Francisco Health Information National Trends Survey by Sexual Orientation and Gender Identity

	All participants, ***n***=1027	SGM, ***n***=130	Non-SGM, ***n***=897	***p***
	***n* (%)**	***n* (%)**	***n* (%)**	
Sociodemographic characteristics				
Age				
Mean (SD)	47.4 (16.7)	40.5 (13.3)	48.4 (16.9)	**<0.001**
Sex at birth				
Male	488 (47.7)	91 (70.5)	397 (44.4)	**<0.001**
Female	536 (52.3)	38 (29.5)	498 (55.6)	
Sexual orientation and gender identity				
Heterosexual men and women	897 (87.3)	—	897 (100)	—
Gay/Lesbian men and women	64 (6.2)	64 (49.2)	—	
Bisexual men and women	39 (3.8)	39 (30.0)	—	
Transgender men and women	14 (1.4)	14 (10.8)	—	
Queer/Other	13 (1.3)	13 (10.0)	—	
Race/ethnicity				
Non-Hispanic White	44 (4.3)	4 (3.1)	40 (4.5)	**<0.001**
Non-Hispanic Black	243 (23.7)	19 (14.6)	224 (25.0)	
Hispanic	365 (35.5)	83 (63.9)	282 (31.4)	
Non-Hispanic Asian/Pacific-Islander	317 (30.9)	13 (10.0)	304 (33.9)	
Other	58 (5.7)	11 (8.5)	47 (5.2)	
Marital status				
Single/Divorced/Separated/Widowed	682 (68.4)	104 (81.9)	578 (66.4)	**<0.001**
Married/Domestic partnership/Living as married	315 (31.6)	23 (18.1)	292 (33.6)	
Education				
Less than HS	236 (23.3)	14 (10.9)	222 (25.1)	**<0.001**
Completed HS	305 (30.2)	28 (21.9)	277 (31.4)	
More than HS	470 (46.5)	86 (67.2)	384 (43.5)	
Employment status				
Unemployed	299 (30.1)	50 (39.4)	249 (28.8)	**<0.001**
Employed	435 (43.9)	60 (47.2)	375 (43.4)	
Retired	137 (13.8)	3 (2.4)	134 (15.5)	
Disabled	121 (12.2)	14 (11.0)	107 (12.4)	
Household income				
<$10k	237 (26.6)	29 (27.9)	208 (26.5)	0.84
$10k to <$20k	274 (30.8)	28 (26.9)	246 (31.3)	
$20k to <$50k	221 (24.8)	27 (26.0)	194 (24.7)	
≥$50k	158 (17.8)	20 (19.2)	138 (17.6)	
Housing unstable	276 (26.9)	64 (49.2)	212 (23.6)	**<0.001**
Born in the United States	482 (47.7)	54 (43.2)	428 (48.4)	0.28
Access to health care				
Health insurance coverage				
None	141 (14.4)	20 (16.1)	121 (14.2)	0.25
Medicaid	167 (17.1)	19 (15.3)	148 (17.3)	
Medicare	318 (32.5)	36 (29.0)	282 (33.0)	
Private	236 (24.1)	27 (21.8)	209 (24.4)	
Other	117 (12.0)	22 (17.7)	95 (11.1)	
Usual place of care	848 (88.3)	110 (88.0)	738 (88.4)	0.90
Times received care in last 12 months (no ER)				
0	204 (19.9)	23 (17.7)	181 (20.2)	0.72
1–2	356 (34.7)	42 (32.3)	314 (35.0)	
3–4	258 (25.1)	35 (26.9)	223 (24.9)	
≥5	209 (20.4)	30 (23.1)	179 (20.0)	

Boldface indicates statistical significance (*p* < 0.05).

ER, emergency room; HS, high school; SD, standard deviation; SGM, sexual and gender minority.

Results of the relationship between SGM status and cancer risk behaviors and beliefs are shown in Table 2. In response to the questions about cancer risk behaviors, 52% of the study population (*n*=493) reported using alcohol frequently, 35% (*n*=363) had a history of smoking at least 100 cigarettes, 25% (*n*=246) reported being current smokers, 23% (*n*=226) reported not exercising during the last month, and 9% (*n*=81) reported using electronic smoking devices. When evaluating these behaviors by SGM status using regression models, SGM participants had significantly higher odds of current smoking (OR=1.72, 95% CI=1.15–2.56) and alcohol use (OR=2.24, 95% CI=1.50–3.34) in unadjusted analysis. In multivariable models, results remained statistically significant for current smoking (OR=1.93, 95% CI=1.24–3.01) and alcohol use (OR=1.69, 95% CI=1.10–2.59).

**Table 2.** Relationship Between Sexual Orientation and Gender Identity and Cancer Risk Behaviors and Cancer Beliefs in the San Francisco Health Information National Trends Survey

	All participants, ***n***=1027	SGM, ***n***=130	Non-SGM, ***n***=897	Unadjusted^a^	Adjusted^a,b^
	***n* (%)**	***n* (%)**	***n* (%)**	**OR (95% CI)**	**OR (95% CI)**
Cancer risk behaviors					
Lifetime smoking, at least 100 cigarettes	363 (35.4)	53 (40.8)	310 (34.6)	1.30 (0.89–1.90)	1.32 (0.87–2.01)
Current smoking	246 (25.3)	44 (34.9)	202 (23.8)	**1.72 (1.15**–**2.56)**	**1.93 (1.24**–**3.01)**
Current use of e-cigarettes or other electronic products	81 (8.6)	16 (12.9)	65 (7.9)	1.72 (0.96–3.08)	1.35 (0.70–2.59)
Alcohol use^c^	493 (51.8)	87 (68.5)	406 (49.3)	**2.24 (1.50**–**3.34)**	**1.69 (1.10**–**2.59)**
Physical inactivity^d^	226 (22.7)	28 (22.1)	198 (22.8)	0.96 (0.61–1.50)	1.01 (0.62–1.63)
Cancer beliefs					
Everything causes cancer	582 (60.0)	74 (57.8)	508 (60.3)	0.90 (0.62–1.31)	0.93 (0.62–1.40)
There is not much you can do to lower your chances of getting cancer	387 (39.9)	43 (34.1)	344 (40.8)	0.75 (0.51–1.12)	1.08 (0.71–1.65)
There are so many recommendations about preventing cancer, it is hard to know which ones to follow	691 (70.2)	81 (64.8)	610 (71.0)	0.75 (0.51–1.12)	0.94 (0.62–1.43)
Cancer is most often caused by behavior or lifestyle	546 (55.7)	68 (54.8)	478 (55.8)	0.96 (0.66–1.41)	1.36 (0.90–2.05)
When I think about cancer, I think about death	632 (63.7)	79 (61.7)	553 (63.9)	0.91 (0.62–1.33)	0.91 (0.61–1.37)

Boldface in estimates indicates statistical significance (*p*<0.05).

^a^ Reference group: non-SGM.

^b^ Models adjusted for age, education, and race/ethnicity.

^c^ Everyday/weekly/monthly use of alcohol; reference group: non-use/once-a-year use.

^d^ Did not exercise during last month in activities such as running, golf, gardening, walking, not work; reference group: exercised in last month.

CI, confidence interval; OR, odds ratio.

A similar pattern in the acceptance of five cancer beliefs was observed in SGM and non-SGM individuals. In descending order of popularity among all participants: “There are so many recommendations about preventing cancer that it is hard to know which ones to follow” (70%, *n*=691); “When I think about cancer, I think about death” (64%, *n*=632); “Everything causes cancer” (60%, *n*=582); “Cancer is most often caused by behavior or lifestyle” (56%, *n*=546); and “There is not much you can do to lower your chances of getting cancer” (40%, *n*=387). Higher proportions of non-SGM participants answered affirmatively to all these statements compared to SGM participants, but no significant differences were observed by SGM status in regression models.

In Table 3 we present the results of the relationship between SGM status and health information-seeking behaviors, preferences, and perceptions. Overall, among study participants, 80% (*n*=801) reported ever having looked for health information or medical topics, with similar frequency in SGM and non-SGM individuals. Of all sources for seeking health information, the preferred source reported by the highest proportion of all participants was the internet (39%, *n*=402), followed by a health care provider (36%, *n*=369), a family/friend/coworker (24%, *n*=251), and written materials (either brochures/pamphlets [21%, *n*=211] or books/magazines/newspapers [21%, *n*=217]). Roughly the same pattern of preferences was observed in all participants irrespective of SGM status, but SGM persons were more likely than non-SGM persons to report a preference for seeking health information online.

**Table 3.** Relationship Between Sexual Orientation and Gender Identity and Health Information Seeking in the San Francisco Health Information National Trends Survey

	All participants, ***n***=1027	SGM, ***n***=130	Non-SGM, ***n***=897	Unadjusted^a^	Adjusted^a,b^
	***n* (%)**	***n* (%)**	***n* (%)**	**OR (95% CI)**	**OR (95% CI)**
Have ever looked for health information/medical topics	801 (80.4)	111 (86.1)	690 (79.6)	**1.75 (1.05**–**2.92)**	1.57 (0.92–2.70)
Preferred sources of health information					
Internet	402 (39.1)	72 (55.4)	330 (36.8)	**2.13 (1.47**–**3.09)**	1.29 (0.86–1.94)
Health care provider	369 (35.9)	56 (43.1)	313 (34.9)	1.41 (0.97–2.05)	1.26 (0.85–1.89)
Family/Friend/Coworker	251 (24.4)	37 (28.5)	214 (23.9)	1.27 (0.84–1.91)	1.32 (0.85–2.06)
Brochure/Pamphlet	211 (20.6)	31 (23.9)	180 (20.1)	1.25 (0.81–1.93)	1.36 (0.85–2.16)
Book/Magazine/Newspaper	217 (21.1)	23 (17.7)	194 (21.6)	0.78 (0.48–1.26)	0.85 (0.51–1.42)
Preferences for getting health information from provider					
Email	425 (41.4)	67 (51.5)	358 (39.9)	**1.60 (1.11**–**2.32)**	1.00 (0.66–1.52)
Brochure/Pamphlet	409 (39.8)	48 (36.9)	361 (40.3)	0.87 (0.59–1.27)	0.96 (0.64–1.45)
Text message	271 (26.4)	44 (33.9)	227 (25.3)	**1.51 (1.02**–**2.24)**	1.24 (0.81–1.89)
Patient portal	120 (11.7)	16 (12.3)	104 (11.6)	1.07 (0.61–1.88)	1.09 (0.60–1.98)
DVD mailed to home	99 (9.6)	18 (13.9)	81 (9.0)	1.62 (0.94–2.80)	1.57 (0.87–2.82)
Source used for getting health information from provider last year					
Email	318 (31.0)	52 (40.0)	266 (29.7)	**1.58 (1.08**–**2.31)**	0.89 (0.58–1.36)
Text message/Instant message application	254 (24.7)	39 (30.0)	215 (24.0)	1.36 (0.91–2.04)	1.22 (0.79–1.89)
Other application on smartphone	136 (13.2)	28 (21.5)	108 (12.0)	**2.01 (1.26**–**3.19)**	1.41 (0.86–2.32)
Video conference	41 (4.0)	8 (6.2)	33 (3.7)	1.72 (0.78–3.80)	1.01 (0.42–2.43)
Fax	29 (2.8)	6 (4.6)	23 (2.6)	1.84 (0.73–4.60)	2.02 (0.73–5.57)
Perceptions most recent health information search					
Lot of effort getting information	466 (49.2)	60 (48.8)	406 (49.2)	1.04 (0.72–1.50)	1.34 (0.90–1.99)
Felt frustrated getting information	413 (42.9)	62 (49.2)	351 (41.9)	1.42 (0.98–2.05)	**1.78 (1.20**–**2.64)**
Had concerns about quality of information	550 (57.0)	81 (64.3)	469 (55.9)	**1.51 (1.03**–**2.20)**	**1.54 (1.03**–**2.31)**
Information was hard to understand	414 (43.6)	47 (39.5)	367 (44.2)	0.82 (0.56–1.20)	1.14 (0.76–1.72)

Boldface in estimates indicates statistical significance (*p*<0.05).

^a^ Reference group: non-SGM.

^b^ Models adjusted for age, education and race/ethnicity.

In the unadjusted analysis, the odds of reporting internet preference in SGM participants were two times as high as in non-SGM participants (OR=2.13, 95% CI=1.47–3.09). In the adjusted analysis, SGM persons still had higher odds of reporting internet use, but results were not statistically significant (OR=1.29, 95% CI=0.86–1.94).

The highest frequency response for preferred method of receiving health information from their provider among both SGM and non-SGM participants was via email (overall 41%, *n*=425), followed by brochure/pamphlet (40%, *n*=409), text message (26%, *n*=271), patient portal (12%, *n*=120), and visual recorded material sent to their homes (10%, *n*=99). In unadjusted models, SGM persons had significantly higher odds of reporting preferences for email (OR=1.60, 95% CI=1.11–2.32) and text message (OR=1.51, 95% CI=1.02–2.24) than non-SGM persons, but in adjusted analyses estimates were not statistically significant (OR=1.00, 95% CI=0.66–1.52; and OR=1.24, 95% CI=0.81–1.89, respectively).

When asked about the sources they have used for receiving health information from their providers in the last year, the same pattern was reported by SGM and non-SGM participants. Email was the most reported source (31%, *n*=318), followed by text message/instant message applications (25%, *n*=254), other applications on smartphones (13%, *n*=136), video conference (4%, *n*=41), and fax (3%, *n*=29). Although SGM participants had significantly higher odds of receiving health information from their provider in the past year via email (OR=1.58, 95% CI=1.08–2.31) and non-instant messaging smartphone applications (OR=2.01, 95% CI=1.26–3.19) compared to non-SGM participants in unadjusted analyses, these results were not statistically significant in multivariable models (OR=0.89, 95% CI=0.58–1.36, for email; and OR=1.41, 95% CI=0.86–2.32, for other applications).

Finally, when asked about their perceptions regarding their most recent search for health information, a high proportion of all participants reported having concerns about the quality of information they obtained (57%, *n*=550), exerting a lot of effort in getting the information (49%, *n*=466), finding the information hard to understand (44%, *n*=414), and feeling frustrated while searching for information (43%, *n*=413). In unadjusted analyses, SGM participants had significantly higher odds of reporting that they were concerned about the quality of information they found (OR=1.51, 95% CI=1.03–2.20). In multivariable analyses, the odds of having concerns about the quality of information (OR=1.54, 95% CI=1.03–2.31) and the odds of feeling frustrated in their search for information (OR=1.78, 95% CI=1.20–2.64) were significantly higher among SGM persons compared to non-SGM participants.

## Discussion

In this study, we evaluated the relationship between SGM status and cancer risk behaviors, cancer beliefs, and health information seeking behaviors, preferences, and perceptions in a multiethnic urban population. Overall, we found that SGM participants were more likely than non-SGM participants to be current smokers and frequent alcohol users, as well as to report frustration while searching health information and concerns about the quality of the information obtained. Despite these differences in perceptions about seeking health information, we found no evidence of differences in behaviors and preferences for health information seeking between SGM and non-SGM individuals, nor did we observe differences in cancer beliefs by SGM status.

The finding that SGM persons engage more than non-SGM persons in cancer risk behaviors such as smoking and alcohol use has been described previously. Using national data, including NHIS and the National Adult Tobacco Survey, researchers have found that sexual minority adults are more likely to use tobacco products and alcohol than heterosexual adults.^13,15^ Other studies ascertaining gender identity information in national (i.e., BRFSS) and regional samples have documented higher prevalence of smoking behaviors and alcohol use among transgender people compared to cisgender people.^41–43^

Also, findings from the 2015 US Transgender Survey estimated that 22% of transgender adults were current smokers (compared to 20% among all US adults in the same year according to the Centers for Disease Control and Prevention), whereas the prevalence of tobacco use among transgender adults who work in the underground economy (i.e., sex work and other criminalized work) was more than 50%, suggesting the complex interplay of social variables that contribute to risk for tobacco use.^44,45^

Several factors have been proposed as possible explanations to the higher prevalence of smoking and alcohol use among SGM persons, including decades of minority-targeted tobacco and alcohol industry advertising and differences in frequency of socializing in bars, locations that have long served as (sometimes the only) safe spaces for SGM individuals to openly gather.^46–48^ Other proposed determinants of these cancer risk behaviors include SES, depression, and chronic stress.^15,49^

In Figure 1, we present a conceptual model of the determinants of cancer outcomes illustrating the relationships between sociodemographic factors that contribute to an individual's identity, the social ecological systems within which the individual exists, and the cyclic nature of psychological and physiological stress, behavioral risk factors, and health information seeking patterns (in short: who a person is, the society in which they live, and what is going on in their mind and body). This model, adapted from the American Heart Association's intersectional transgender multilevel minority stress model,^12^ draws from theories of the social determinants of health to help us understand how SGM minority status and experiences of SOGI-based stigma may influence risk behaviors like smoking and alcohol use and how psychosocial factors like stigmatization burden may contribute to feelings of frustration when seeking health information and anxiety about the quality of the information obtained.^5,50–52^

In this study population, a high prevalence of health information seeking was observed (≥80%). Overall, study participants preferred digital means searching for health information (internet) and receiving health information from their providers (email), and higher proportions of SGM individuals—who were 8 years younger on average than non-SGM participants in this sample—reported preferences for these digital methods. These findings are consistent with previous research and suggest that irrespective of SGM status, participants relied on using digital technology for accessing and exchanging health information, more than other in-person, less private and less instantaneous methods.^53,54^ These results also present specific challenges and opportunities for health care providers and public health organizations in their efforts to improve SGM health.

Many sources of health information are available on the internet, including numerous options not necessarily guided by science, and these can create confusion for SGM and non-SGM persons alike who utilize this information to make health-related choices.^55^ On the other hand, providing information that people can access whenever they need, in different media (e.g., audio, video, text), and free of cost can be hugely beneficial for people, especially for those belonging to the most marginalized and disadvantaged sociodemographic groups. Thus, to improve the quality of health information that SGM persons and other vulnerable populations receive on the internet, efforts should be directed toward community-partnered development of health information content, and the preferences of SGM and multiply marginalized communities must be sought and considered in strategies for message dissemination (e.g., what websites and social media they prefer to look to for health information, what organizations they trust and follow on social media).

In our analysis, smoking remained significantly elevated even after adjustment for education as a proxy for SES. Recent research also found that SGM persons are more likely than non-SGM persons to be exposed to tobacco-related marketing and social media, and exposure to these messages was associated with tobacco use.^56^ With the high preference for seeking information online and for digital communication with providers observed in this study (i.e., email, text messaging apps, video conference), SGM-tailored smoking cessation interventions that incorporate these strategies and target culturally specific barriers, including immediate and chronic stressors, should be explored in this community.^57^

In the evaluation of participants' perceptions during the most recent health information search, we found that SGM participants had significantly higher odds of experiencing frustration and of having concerns about the quality of information than non-SGM participants. Prior research has documented increased wariness of health misinformation among SGM individuals, but to our knowledge, no studies have previously described increased feelings of frustration when searching for health information.^58^ Frustration may be a substantial barrier for SGM persons in obtaining health information and following recommendations, as these feelings tend to discourage people from further searches.^59^

This finding made us consider a set of key questions that remain unanswered. What are the drivers of frustration among SGM people while seeking health information? Do SGM individuals have difficulties in accessing the information? Do SGM persons find the information they need? Is the information that SGM people look for available? Is the content that they find inclusive to all? Do SGM individuals feel satisfied that the information they have found suits their needs? Is it trustworthy, and do SGM individuals perceive it to be trustworthy? Future research into these topics should consider employing qualitative or mixed methods, as they may be better suited to these research questions.

This study has several limitations. First, despite following current best practices for measuring SOGI data, misclassification of SGM individuals could still be present in this study. Although participants were able to complete the survey themselves, minimizing the likelihood of underreported SGM status, not all questionnaires were self-administered, not all participants may have been willing to disclose their SGM status, and other barriers to perfect measurement may still exist. We cannot rule out the possibility that non-SGM individuals may be unfamiliar with the concepts and language of SOGI and erroneously selected one of the SGM categories.^29^ Also, we only assessed one aspect of sexual orientation (i.e., identity), and did not consider behavior or attraction that is also recommended as best practice.^28^

Similarly, we assessed gender identity and concordance with sex assigned at birth, but we did not ask participants about their gender expression, others' perceptions of their gender, or differences in sex development (also described as intersex status). Second, misclassification of the different outcomes is also possible, especially if people feel that a particular response may be more acceptable than others (e.g., social desirability bias on cancer risk behaviors questions). Third, we focused our sampling efforts to include specific populations by our choice of specific neighborhoods and recruitment sites and by prespecifying sample proportions according to race, ethnicity, and language spoken. We did not also prespecify sample proportions by SOGI status, so these subgroups remained small in the sample. To increase the statistical power of our SGM analyses, we consolidated all SGM persons into a single group.

This data aggregation could obscure or reverse the true relationships between specific SGM subgroups and the outcomes under study (i.e., Simpson's paradox).^60^ Also, prior research has detected differences in behaviors and sociodemographic composition within SGM subgroups, and experts recommend analyzing them separately when possible.^28^ In this study, compared to all SGM subgroups, bisexual and transgender individuals reported higher prevalence of cancer risk behaviors and bisexual individuals reported higher prevalence of fatalistic views about cancer (Supplementary Table S1). Fourth, despite these efforts, our analyses were constrained by a modest sample size, as evidenced in the wide CIs of some estimates. Fifth, in our interest to study a hard-to-reach population, we employed a non-probabilistic sampling method, but we did not include important information throughout the process (i.e., seeds, connections) that would allow us to use a more appropriate analytic strategy for this type of sampling design (as in respondent-driven sampling).^30,61^

Lastly, San Francisco is a culturally, economically, politically, and demographically unique city, and these results may not be generalizable to multiethnic SGM populations in all other major US cities, nor in even more dissimilar settings. We expect the experiences of SGM residents of San Francisco to be more similar to those of SGM persons in other dense, politically and socially liberal, multiethnic US cities with high income inequality than to those of SGM persons in rural, racially and ethnically homogenous, or politically and socially conservative parts of the country.

However, this study's findings of increased risk of current smoking and alcohol use, in addition to concerns about health information quality among SGM individuals are consistent with prior research. Our finding of increased feelings of frustration among SGM individuals when searching for health information is plausible in any setting, particularly if widespread hetero- and cissexism are contributing mechanisms of this frustration. As we move increasingly into online spaces where we engage in increasingly shared cultural experiences—particularly among younger SGM adults—some common barriers to generalizability may be further minimized.

## Conclusions

In this diverse urban population, a preference for the use of digital communication technologies for accessing and exchanging health information was reported, irrespective of SGM status. Funding for the development and expansion of tobacco cessation interventions that target SGM persons must be prioritized. New and existing uses of delivering these interventions via online platforms, instantaneous messaging applications, and other digital technologies that promote access should be evaluated for scalability. Additionally, community-partnered program development and qualitative research on the drivers of experiencing frustration while seeking health information will be critical to understanding and overcoming common barriers in health information seeking among SGM communities, with great implications for SGM health and access to care and knowledge.

Finally, although progress has been made in collecting SOGI data and including SGM people in population health research, still no major national population health surveys capture gender expression—a key factor in experiences of gender-related stigma—nor do they measure populations with differences in sex development. Sustained efforts are needed until all major national datasets and research endeavors capture complete SOGI information regularly, following up-to-date best practices.

## Supplementary Material

Supplemental data

## Abbreviations Used

BRFSS: Behavioral Risk Factor Surveillance System
CI: confidence interval
ER: emergency room
HINTS: Health Information National Trends Survey
HIV: human immunodeficiency virus
HS: high school
NHIS: National Health Interview Survey
OR: odds ratio
SD: standard deviation
SES: socioeconomic status
SF CAN: San Francisco Cancer Initiative
SF-HINTS: San Francisco Health Information National Trends Survey
SGM: sexual and gender minority
SOGI: sexual orientation and gender identity
