# Supplementary material for: Cancer Risk Behaviors, Cancer Beliefs, and Health Information Seeking Among Under-Represented Populations in San Francisco: Differences by Sexual Orientation and Gender Identity
Source: Health Equity. 2022 Sep 1;6(1):669–80. doi: 10.1089/heq.2022.0013 (PMC9536334; doi:10.1089/heq.2022.0013)
Supplement: Supplemental data [file Supp_TableS1.docx]

| **Supplemental Table. Cancer risk behaviors, cancer beliefs and health information seeking by sexual orientation and gender identity in the San Francisco Health Information National Trends Survey, n (%)** | | | | | | |
| --- | --- | --- | --- | --- | --- | --- |
|  | **All participants, n=1027** | **Non-SGM, n=897** | **Gay/Lesbian,**  **n=64** | **Bisexual,**  **n=39** | **Transgender,**  **n=14** | **Queer/Other,**  **n=13** |
| **Cancer risk behaviors** |  |  |  |  |  |  |
| Lifetime smoking, at least 100 cigarettes | 363 (35.4) | 310 (34.6) | 24 (37.5) | 19 (48.7) | 5 (35.7) | 5 (38.5) |
| Current smoking | 246 (25.3) | 202 (23.8) | 19 (29.7) | 17 (48.6) | 4 (28.9) | 4 (30.8) |
| Current use of e-cigarettes or other electronic products | 81 (8.6) | 65 (7.9) | 3 (4.9) | 8 (22.2) | 3 (21.4) | 2 (15.4) |
| Alcohol use ^a^ | 493 (51.8) | 406 (49.3) | 40 (64.5) | 28 (73.7) | 11 (78.6) | 8 (61.5) |
| Physical inactivity ^b^ | 226 (22.7) | 198 (22.8) | 11 (17.5) | 10 (26.3) | 5 (38.5) | 2 (15.4) |
| **Cancer beliefs** |  |  |  |  |  |  |
| Everything causes cancer | 582 (60.0) | 508 (60.3) | 34 (54.0) | 25 (64.1) | 6 (46.2) | 9 (69.2) |
| There is not much you can do to lower your chances of getting cancer | 387 (39.9) | 344 (40.8) | 19 (30.2) | 17 (44.7) | 5 (38.5) | 2 (16.7) |
| There are so many recommendations about preventing cancer, it is hard to know which ones to follow | 691 (70.2) | 610 (71.0) | 38 (61.3) | 27 (69.2) | 9 (64.3) | 7 (70.0) |
| Cancer is most often caused by behavior or lifestyle | 546 (55.7) | 478 (55.8) | 33 (54.1) | 25 (65.8) | 4 (28.6) | 6 (54.6) |
| When I think about cancer, I think about death | 632 (63.7) | 553 (63.9) | 36 (58.1) | 29 (74.4) | 6 (42.9) | 8 (61.5) |
| **Health information seeking** |  |  |  |  |  |  |
| Have ever looked for health information/medical topics | 801 (80.4) | 690 (79.6) | 57 (90.5) | 32 (82.1) | 12 (85.7) | 10 (76.9) |
| **Preferred source of health information** |  |  |  |  |  |  |
| Internet | 402 (39.1) | 330 (36.8) | 38 (59.4) | 22 (56.4) | 5 (35.7) | 7 (53.9) |
| Healthcare provider | 369 (35.9) | 313 (34.9) | 26 (40.6) | 19 (48.7) | 8 (57.1) | 3 (23.1) |
| Family/Friend/Coworker | 251 (24.4) | 214 (23.9) | 16 (25.0) | 11 (28.2) | 5 (35.7) | 5 (38.5) |
| Brochure/Pamphlet | 211 (20.6) | 180 (20.1) | 16 (25.0) | 12 (30.8) | 1 (7.1) | 2 (15.4) |
| Book/Magazine/Newspaper | 217 (21.1) | 194 (21.6) | 14 (21.9) | 7 (18.0) | 1 (7.1) | 1 (7.7) |
| **Preferences for getting health information from provider** |  |  |  |  |  |  |
| Email | 425 (41.4) | 358 (39.9) | 36 (56.3) | 20 (51.3) | 5 (35.7) | 6 (46.2) |
| Brochure/Pamphlet | 409 (39.8) | 361 (40.3) | 19 (29.7) | 19 (48.7) | 6 (42.9) | 4 (30.8) |
| Text message | 271 (26.4) | 227 (25.3) | 18 (28.1) | 16 (41.0) | 6 (42.9) | 4 (30.8) |
| Patient portal | 120 (11.7) | 104 (11.6) | 6 (9.4) | 6 (15.4) | 2 (14.3) | 2 (15.4) |
| DVD mailed to home | 99 (9.6) | 81 (9.0) | 8 (12.5) | 7 (18.0) | 2 (14.3) | 1 (7.7) |
| **Source used for getting health information from provider last year** |  |  |  |  |  |  |
| Email | 318 (31.0) | 266 (29.7) | 30 (46.9) | 10 (25.6) | 5 (35.7) | 7 (53.9) |
| Text message/Instant message application | 254 (24.7) | 215 (24.0) | 15 (23.4) | 12 (30.8) | 6 (42.9) | 6 (46.2) |
| Other application on smartphone | 136 (13.2) | 108 (12.0) | 13 (20.3) | 6 (15.4) | 3 (21.4) | 6 (46.2) |
| Video conference | 41 (4.0) | 33 (3.7) | 3 (4.7) | 2 (5.1) | 3 (21.4) | 0 (0) |
| Fax | 29 (2.8) | 23 (2.6) | 3 (4.7) | 2 (5.1) | 0 (0) | 1 (7.7) |
| **Perceptions most recent health information search** |  |  |  |  |  |  |
| Lot of effort getting information | 466 (49.2) | 406 (49.2) | 29 (45.3) | 18 (46.2) | 7 (50.0) | 6 (46.2) |
| Felt frustrated getting information | 413 (42.9) | 351 (41.9) | 27 (42.2) | 23 (59.0) | 4 (28.6) | 8 (61.5) |
| Had concerns about quality of information | 550 (57.0) | 469 (55.9) | 34 (53.1) | 29 (74.4) | 8 (57.1) | 10 (76.9) |
| Information was hard to understand | 414 (43.6) | 367 (44.2) | 17 (26.6) | 17 (43.6) | 3 (21.4) | 10 (76.9) |
| ^a^ Everyday/weekly/monthly use of alcohol  ^b^ Did not exercise during last month in activities such as running, golf, gardening, walking, not work  SGM: sexual and gender minority | | | | | | |
